# Regnase-1 downregulation promotes pancreatic cancer through myeloid-derived suppressor cell-mediated evasion of anticancer immunity

**DOI:** 10.1186/s13046-023-02831-w

**Published:** 2023-10-09

**Authors:** Junya Okabe, Takahiro Kodama, Yu Sato, Satoshi Shigeno, Takayuki Matsumae, Kazuma Daiku, Katsuhiko Sato, Teppei Yoshioka, Minoru Shigekawa, Masaya Higashiguchi, Shogo Kobayashi, Hayato Hikita, Tomohide Tatsumi, Toru Okamoto, Takashi Satoh, Hidetoshi Eguchi, Shizuo Akira, Tetsuo Takehara

**Affiliations:** 1 Department of Gastroenterology and Hepatology, Osaka University Graduate School of Medicine, Suita, Japan; 2 Department of Gastroenterological Surgery, Osaka University Graduate School of Medicine, Suita, Japan; 3 Department of Microbiology, Juntendo University School of Medicine, Tokyo, Japan; 4 Institute for Advanced Co-Creation Studies, Research Institute for Microbial Diseases, Osaka University, Suita, Japan; 5 Department of Immunology, Graduate School of Medical and Dental Sciences, Tokyo Medical and Dental University, Tokyo, Japan; 6 Laboratory of Host Defense, World Premier Institute Immunology Frontier Research Center, Osaka University, Suita, Japan; 7 Department of Host Defense, Research Institute for Microbial Diseases, Osaka University, Suita, Japan

**Keywords:** PDAC, MDSCs, Inflammation, CTL, CD11b

## Abstract

**Background:**

Pancreatitis is known to be an important risk factor for pancreatic ductal adenocarcinoma (PDAC). However, the exact molecular mechanisms of how inflammation promotes PDAC are still not fully understood. Regnase-1, an endoribonuclease, regulates immune responses by degrading mRNAs of inflammation-related genes. Herein, we investigated the role of Regnase-1 in PDAC.

**Methods:**

Clinical significance of intratumor Regnase-1 expression was evaluated by immunohistochemistry in 39 surgically-resected PDAC patients. The functional role of Regnase-1 was investigated by pancreas-specific Regnase-1 knockout mice and Kras-mutant Regnase-1 knockout mice. The mechanistic studies with gene silencing, RNA immunoprecipitation sequencing (RIP-seq) and immune cell reconstitution were performed in human/mouse PDAC cell lines and a syngeneic orthotopic tumor transplantation model of KrasG12D-mutant and Trp53-deficient PDAC cells.

**Results:**

Regnase-1 expression was negatively correlated with the clinical outcomes and an independent predictor of poor relapse-free and overall survival in PDAC patients. Pancreas-specific Regnase-1 deletion in mice promoteed pancreatic cancer with PMN-MDSC infiltration and shortened their survival. A syngeneic orthotopic PDAC model exhibited that Regnase-1 downregulation accelerated tumor progression via recruitment of intratumor CD11b^+^ MDSCs. Mechanistically, Regnase-1 directly negatively regulated a variety of chemokines/cytokines important for MDSC recruitment and activation, including CXCL1, CXCL2, CSF2, and TGFβ, in pancreatic cancer cells. We subsequently showed that IL-1β-mediated Regnase-1 downregulation recruited MDSCs to tumor sites and promoted pancreatic cancer progression via mitigation of cytotoxic T lympohocytes-mediated antitumor immunity.

**Conclusions:**

IL-1b-mediated Regnase-1 downregulation induces MDSCs and promotes pancreatic cancer through the evasion of anticancer immunity.

**Supplementary Information:**

The online version contains supplementary material available at 10.1186/s13046-023-02831-w.

## Background

Pancreatic cancer is the fourth leading cause of cancer deaths and is estimated to become the second most common cause within 10 years [1]. The prognosis of pancreatic cancer patients is dismal, with a 5-year survival rate of 4.2% [2]. Development of effective therapeutics and early detection methods is urgently needed. Chronic pancreatitis (CP) is known to be an important risk factor for pancreatic cancer [3]. Previous studies showed that a combination of inflammation and Kras mutation strongly accelerated the development and progression of pancreatic cancer [4]. However, the exact molecular mechanisms of how inflammation promotes pancreatic cancer are still not fully clarified.

Innate immunity responds to various factors, such as infection and tissue damage, and plays an important role in the development and control of diseases [5, 6]. Inflammatory responses induced by innate immune cells such as macrophages and dendritic cells are important for maintaining the homeostasis of organisms, such as the elimination of pathogens [6]. However, excessive immune responses and chronic inflammation lead to the development of autoimmune diseases and chronic inflammatory diseases [7]. For this reason, pro-inflammatory cytokines are strictly regulated by transcriptional and post-transcriptional mechanisms [8]. Recent studies have shown that Regnase-1, an endoribonuclease, acts as a brake on inflammation by degrading the mRNA of various inflammatory cytokines [9, 10]. Indeed, Regnase-1 knockout mice spontaneously developed autoimmune disease, revealing the importance of Regnase-1 as a regulator of inflammation [9, 11]. Recent reports have shown that Regnase-1 is also involved in cancer biology through various mechanisms, such as the regulation of apoptosis and metastatic potential [12–15]. However, the role of Regnase-1 in pancreatic cancer remains unclear.

In this study, we hypothesized that Regnase-1 may serve as an important link between inflammation and pancreatic cancer progression. To study this hypothesis, we investigated the role of Regnase-1 using human pancreatic ductal adenocarcinoma (PDAC) specimens, genetically engineered mice, and a syngeneic orthotopic tumor transplantation model. We showed here that intratumor Regnase-1 expression was negatively correlated with poor clinical outcomes in pancreatic cancer patients. Mechanistically, we showed that inflammatory cytokine-induced Regnase-1 downregulation promoted pancreatic cancers via myeloid-derived suppressor cell (MDSC)-mediated evasion of antitumor immunity in mice. Our study revealed the novel mechanistic link between inflammation and PDAC progression and may pave the way for the development of therapeutics targeting inflammation-mediated cancer progression in PDAC patients.

## Methods

### GEMM

C57BL/6 J mice were obtained from The Jackson Laboratory; Pdx1-Cre transgenic mice and KrasLSLG12D knock-in mice were obtained from the Mouse Models of Human Cancer Consortium (National Cancer Institute-Frederick, Bethesda, Maryland). Regnase-1 floxed mice were obtained from the Institute of Microbiology, Osaka University, Japan. KrasLSLG12D transgenic mice or Regnase-1 floxed mice were crossed with Pdx1-Cre mice to generate Pdx1-Cre KrasLSLG12D mice or Pdx1-Cre Regnase-1 fl/ + mice. The resulting mice were then crossed to generate KrasLSLG12D Regnase-1 fl/fl (WT) mice, Pdx1-Cre KrasLSLG12D (PK) mice, Pdx1-Cre Regnase-1 fl/fl (PR) mice, and Pdx1-Cre KrasLSLG12D Regnase-1 fl/fl (PKR) mice.

### Syngeneic orthotopic model

BL6/J mice were anesthetized with midazolam, butorphanol, and medetomidine prior to surgical resection to reduce animal distress, and every effort was made to reduce animal distress. We previously established murine pancreatic cancer cell lines named KPCs from the genetically engineered pancreatic cancer mouse model ElaCre Kras^LSLG12D^trp53^fl/fl^ EYFP^Tg/Tg^ mice [16]. A total of 1.0 × 10^5^ KPC cells were suspended in Matrigel (356,230, Corning, NY, USA) and injected into the tail of the mouse pancreas. Twenty-one days after ipsilateral injection, mice were euthanized. The dissected tumors were weighed and divided for each analysis.

BE0075-1, a fully neutralizing mAb recognizing Gr-1, and control IgG were obtained from Bioxcell. Starting 1 day after syngeneic transplantation, anti-Gr-1 antibody was injected intraperitoneally at 100 μg/animal three times a week. Controls were treated with vehicle; after 3 weeks of treatment, mice were euthanized and dissected, and tumor weights were determined. Part of the tumor was used for each analysis.

BE0061, a fully neutralizing mAb recognizing CD8a, and control IgG were obtained from Bioxcell. Starting 1 day after syngeneic transplantation, anti-CD8a antibody or IgG was injected intraperitoneally at 300 μg/animal twice a week. After 3 weeks of treatment, mice were euthanized and dissected, tumor weights were determined, and part of the tumor was used for each analysis.

CD11b-positive cells were extracted from tumors generated by syngeneic transplantation by positive selection using CD11b magnetic cell separation system beads (Miltenyi Biotec, Germany). A total of 1.0 × 10^5^ KPC cells or an equal number of KPC cells plus 1.0 × 10^4^ extracted CD11b-positive cells were suspended in Matrigel (356,230, Corning, NY, USA) and injected into the tail of the mouse pancreas. Twenty-one days after ipsilateral injection, mice were euthanized and dissected, tumors were weighed, and part of the tumor was used for each analysis.

### Tissue preparation

All mice were euthanzied and pancreatic samples were collected for study. Each pancreas was quickly removed, weighed, and either snap frozen for molecular analysis or fixed in 10% neutral phosphate buffered formalin for histological analysis.

### Histologic and immunohistochemical analysis

Pancreatic tissues were stained with standard H&E preparations. Immunohistochemical analysis of paraffin-embedded pancreatic tissue was performed with an antibody specific for CD11b (Cell Signaling Technology) and CD8a (Proteintech). Immunostained tissues were photographed using a SLIDEVIEW VS200 (Olympus), and the average number of stained cells per field (total 3 fields, 2.6mm^2^ per field) was quantified.

### qPCR analysis

RNA isolation and quantitative PCR were performed. Total RNA was extracted from cell lines or pancreatic tissue using the RNeasy Mini Kit (Qiagen) according to the manufacturer's instructions, and cDNA was synthesized by reverse transcription as described previously. Quantitative PCR was performed using a Quant Studio 6 Flex real-time PCR system (Thermo Fisher Scientific) with TaqMan probes. Relative gene expression levels were determined by the ΔΔCT method and normalized to Actb expression levels. Detailed information on the probes (Thermo Fisher Scientific) is provided in Supplementary Table 1.

### Western blot analysis

Samples of pancreatic tissue or harvested cells were incubated in lysis buffer (1% Nonidet P-40, 0.5% sodium deoxycholate, 0.1% SDS, protease inhibitor cocktail [Nacalai Tesque], phosphatase inhibitor cocktail [Nacalai Tesque], and PBS [pH 7.4]). Equal amounts of protein were separated by electrophoresis and subjected to Western blotting. The following antibodies were used as primary antibodies: anti-Regnase-1 (manufactured by Sigma-Aldrich) and anti-ACTB (Sigma-Aldrich). Raw blot images of WB were provided in Supplementary Fig. 1.

### Cell culture

The human pancreatic cancer tumor cell lines Panc-1 and MiaPaCa2 were obtained from the American Type Culture Collection cell bank and cultured in DMEM (Nacalai Tesque). Mouse pancreatic cancer cell lines were established in the past at our laboratory as described above [16]. All cells were cultured at 37 °C in a humidified atmosphere containing 5% CO_2_ and were confirmed to be free of pathogens and mycoplasma. Recombinant IL-1β protein was obtained from Peprotech. Recombinant IL-1β protein was administered to human pancreatic cancer cell lines (Panc-1, MiaPaCa2) at 100 ng/ml, and RNA/protein was harvested 1 h later.

### Cell transfection with siRNA

Silencer Select siRNA against human Regnase-1 was obtained from Thermo Fisher Scientific. Two independent siRNAs against Regnase-1 (#1 and #2) were used. Cells were transfected by a reverse transfection method. First, cells were transfected with siRNA (10 nM) using Lipofectamine RNAiMAX (Thermo Fisher Scientific) according to the manufacturer's instructions. Briefly, cells were plated in 12-well plates. After plating, either Silencer Select negative control siRNA or target siRNA mixed with Lipofectamine RNAiMAX was added to the plates on the same day. The siRNA was added to the plates on the same day. Cells were used for experiments three to five days after transfection. Detailed siRNA information is provided in Supplementary Table 1.

### Generation of KO cell line by CRISPR/Cas9

Murine pancreatic cancer cells (KPCs) were transfected with vectors expressing Cas9 and gRNA targeting Regnase-1 using Lipofectamine 3000. After transfection, multiple single clones were obtained by the limited dilution method. Regnase-1 deletion was confirmed by western blot.

### RIP-seq

RIP-seq was performed according to the methods reported previously [17]. Briefly, wild-type Regnase-1 vector or mutant (D141N) Regnase-1 vector lacking RNase activity was transiently transfected into Regnase-1-deficient Panc-1 cells. RNA–protein complexes were immunoprecipitated and extracted RNA was sequenced on a HiSeq 2000 system (Illumina) according to the manufacturer's instructions. The resulting set of trimmed reads was then mapped against the human genome. We compared the normalized mRNA read counts of each gene between cells with wild-type Regnase-1 vector or mutant (D141N) Regnase-1 vector (Supplementary Table 2). Both wild-type and mutant Regnase-1 proteins can bind to direct targets because of the same binding capacity. On the other hand, WT Regnase-1 protein can degrade the bound mRNA but mutant Regnase-1 protein cannot. Therefore, genes showing higher read counts in cells with mutant Regnase-1 protein compared to those in cells with WT Regnase-1 protein suggest the direct targets of Regnase-1.

### Human samples

Thirty-nine patients who underwent surgery for resectable pancreatic cancer at the Department of Gastroenterological Surgery, Osaka University Graduate School of Medicine between February 2011 and April 2018 were studied. Patients who received preoperative treatment such as radiotherapy or chemotherapy were excluded. Immunostaining with antibodies specific for Regnase-1 and CD11b was performed on sections containing cancerous areas, and the intensity of Regnase-1 staining and the number of stained cells were scored and compared with the number of CD11b-positive cells, prognosis, and recurrence-free survival rate.

### Statistical analysis

Prism 9.0.1 software (GraphPad) was used for statistical analysis. Data are presented as the mean ± SD, and two-tailed Student's t test was used to evaluate differences between the two groups. One-way analysis of variance and Tukey's post hoc test were used to compare differences among three or more groups. Survival data were analyzed using the log-rank test, with a *P* value of less than 0.05 considered significant.

### Study approval

All animal procedures were performed in accordance with the Animal Care and Use Guidelines of Osaka University and approved by the Ethics Committee of the Osaka University Graduate School of Medicine. Approval was obtained from the Ethics Committee of the Osaka University Graduate School of Medicine. The use of excised human specimens was approved by the Ethics Committee of the Osaka University Graduate School of Medicine. Approval was obtained from the Ethics Committee of Osaka University Graduate School of Medicine (protocol 17,160), and written informed consent was obtained from all patients. The study design adhered to the tenets of the Declaration of Helsinki.

## Result

### Regnase-1 expression is negatively correlated with the clinical outcomes and intratumor infiltration of CD11b^+^ cells in PDAC patients

**Fig. 1:**
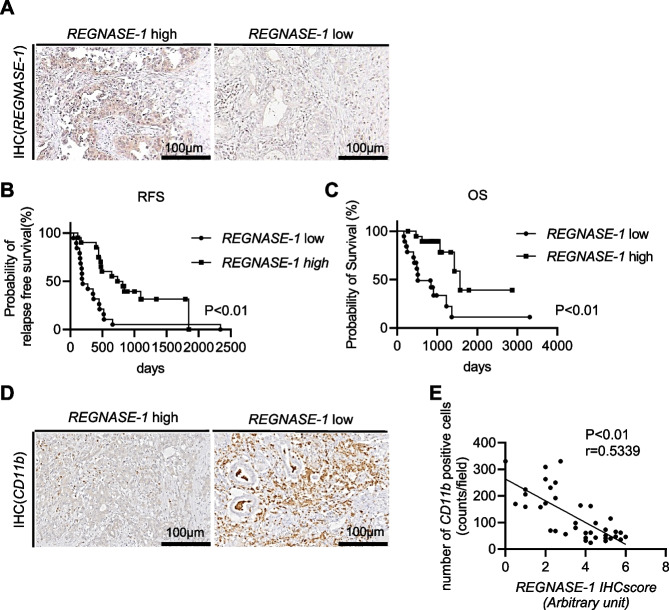
Regnase-1 expression is negatively correlated with the clinical outcomes and intratumor infiltration of CD11b^+^ cells in PDAC patients. **A** Representative images of Regnase-1 staining of pancreatic tumors from pancreatic cancer patients. **B-C** Thirty-nine pancreatic cancer patients were classified into two groups according to the Regnase-1 immunostaining scores in pancreatic tumors. Kaplan–Meier curves of recurrence-free survival (RFS) (**B**) and overall survival (OS) (**C**) in each group. **D** Representative images of CD11b staining of pancreatic tumors in pancreatic cancer patients. **E** Correlation between Regnase-1 immunostaining scores and CD11b.^+^ cell counts of pancreatic tumors in pancreatic cancer patients. The Pearson product-moment correlation coefficient was used to determine the correlation coefficient. Scale bars: 100 μm (insets)

First, we aimed to clarify the clinical importance of Regnase-1 in PDAC patients. To this end, we examined the Regnase-1 protein levels in the tumor sites by immunohistochemistry in 39 patients who underwent curative surgical resection (Fig. 1A). The clinical background of these patients is shown in Supplementary Table 3. All patients were in stage 1 or 2, and the median recurrence-free and overall survival times were 14.0 and 47.7 months, respectively. Patients were stratified into 2 groups based on the median IHC scores (Supplementary Table 3). The low Regnase-1 expression group showed significantly shorter recurrence-free survival and overall survival than the high Regnase-1 expression group (Fig. 1B, C). In addition, univariate and multivariate Cox proportional hazard analyses showed that low levels of Regnase-1 and carcinoembryonic antigen (CEA) were significantly associated with poor relapse-free and overall survival (Table 1). We next evaluated immune cells including myeloid and lymphoid cells infiltrating into the tumors and found strong infiltration of CD11b^+^ cells in the tumors of the low Regnase-1 expression group with a significant negative correlation between Regnase-1 expression and the number of infiltrated CD11b^+^ cells (Fig. 1D, E, Supplementary Fig. 2A, B, Supplementary Table 3). These findings suggested that the downregulation of Regnase-1 may correlate with tumor progression and intratumor recruitment of CD11b^+^ cells in human PDAC.

**Table1:** Factors associated with relapse free survival and overall survival of pancreatic ductal adenocarcinoma patients after curative resection

		Relapse free survival (RFS)				Overall survival (OS)			
Factor	Unit	Univariate analysis		Multivariate analysis		Univariate analysis		Multivariate analysis	
		Hazard ratio (95% CI)	*p* value	Hazard ratio (95% CI)	*p* value	Hazard ratio (95% CI)	*p* value	Hazard ratio (95% CI)	*p* value
Age	Years		0.749				0.537		
Gender	Male/ Female		0.434				0.598		
Tumor size	mm	1.07 (1.02–1.12)	0.006		0.123		0.075		
Tumor pathology	tub/ non-tub		0.350				0.229		
Regnase-1 IHC score		0.64 (0.50–0.81)	< 0.001	0.66 (0.51–0.85)	0.002	0.67 (0.52–0.85)	0.001	0.64 (0.49–0.83)	0.001
CEA	ng/mL	1.14 (1.03–1.25)	0.018	1.15 (1.03–1.26)	0.013	1.18 (1.05–1.31)	0.008	1.20 (1.06–1.34)	0.004
CA19-9	U/mL		0.133						

### Pancreas-specific Regnase-1 deletion promotes pancreatic oncogenesis with polymorphonuclear-myeloid-derived suppressor cell (PMN-MDSC) infiltration in mice

**Fig. 2:**
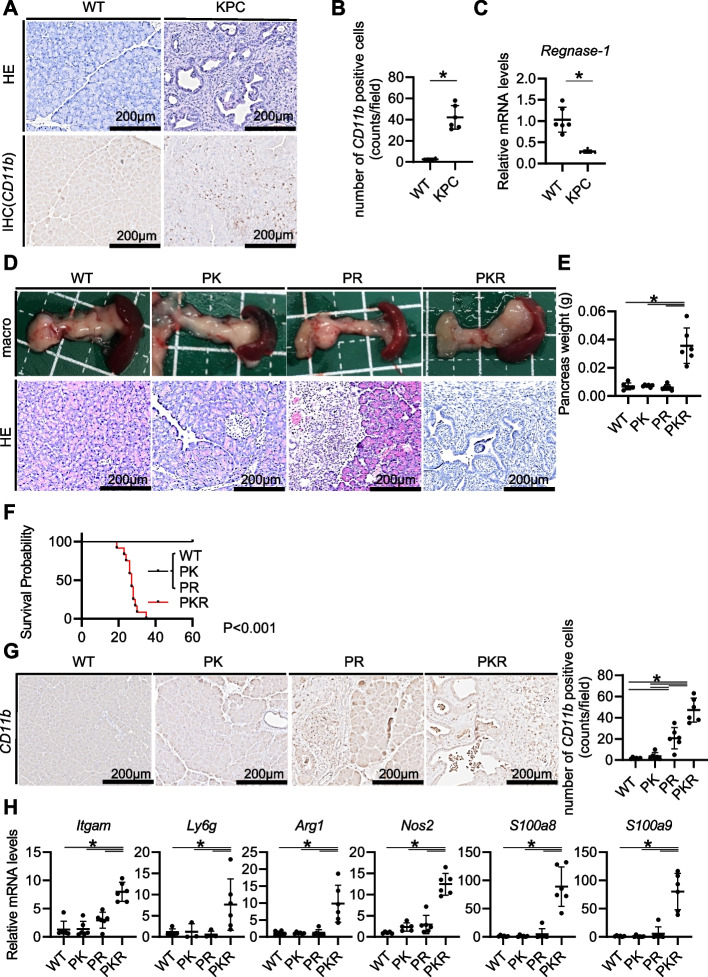
Pancreas-specific Regnase-1 deletion promotes pancreatic oncogenesis with PMN-MDSC infiltration in mice. **A-C** Pancreatic phenotypes were examined in wild-type (WT) mice and pancreas-specific Kras- and Tp53-mutant (KPC) mice at 6 weeks of age. Representative images of HE staining (top) and CD11b immunostaining (bottom) in the pancreas of WT mice and pancreatic tumors of KPC mice (**A**). Number of CD11b-positive cells (**B**) (*N* = 6 per group) and relative *Regnase-1* (*Zc3h12a*) mRNA levels (**C**) (*N* = 6 per group) in the pancreas of WT mice and pancreatic tumors of KPC mice. **D-H** Pancreatic phenotypes were examined in WT mice, pancreas-specific Regnase-1 knockout (PR) mice, pancreas-specific Kras-mutant (PK) mice, and pancreas-specific Kras-mutant Regnase-1 knockout (PKR) mice at 4 weeks of age. Macroscopic image (top) and HE staining (bottom) of the pancreas (**D**). Pancreatic weights in each group (**E**) (*N* = 6 per group). Kaplan–Meier curves of overall survival in each group (**F**) (*N* = 12 per group). Representative images of CD11b immunostaining (**G **left) and the number of CD11b-positive cells (**G** right) in each group (*N* = 6 per group). Relative mRNA levels (**H**) of *Itgam*, *Ly6g*, *Arg1*, *Nos2*, *S100a8*, and *S100a9* in pancreatic tissue (*N* = 6 per group). One-way analysis of variance followed by Tukey's post hoc test was used to compare differences between the four groups. *: *P* < 0.05, Scale bars: 200 μm (insets)

Next, we investigated murine pancreatic tumors that spontaneously developed in aged pancreas-specific KrasG12D mutant and Tp53 knockout mice (KPC mice). Significant downregulation of Regnase-1 expression with strong CD11b^+^ cell infiltration was observed in the tumors of the KPC mice compared to the normal pancreatic tissues (Fig. 2A-C). To elucidate the role of Regnase-1 downregulation in pancreatic cancer, we generated pancreas-specific Regnase-1 knockout mice and crossed them with pancreas-specific KrasG12D mutant mice (PK mice). Regnase-1 knockout mice developed normally but spontaneously developed focal pancreatitis, as shown by HE staining (Fig. 2D, Supplementary Fig. 3). Importantly, pancreas-specific Regnase-1 knockout and KrasG12D mutant mice (PKR mice) spontaneously developed PDAC, and all died within 40 days of age (Fig. 2D-F). PKR mice showed strong infiltration of CD11b^+^ cells in the pancreatic tumor sites (Fig. 2G). We then examined a variety of inflammatory cytokines/chemokines and found significant upregulation of several genes expressed in MDSCs, including *Arg1*, *Nos2*, *S100a8*, and *S100a9,* together with the PMN-MDSC markers *Itgam* and *Ly6g* (Fig. 2H, Supplementary Fig. 4). Flow cytometry showed that most of CD11b^+^ cells in the PKR mice were Ly6g + Ly6c^low^ cells (Supplementary Fig. 5). Taken together, these findings suggested that pancreas-specific Regnase-1 loss may promote PDAC oncogenesis with PMN-MDSC infiltration in mice.

### Regnase-1 deletion in pancreatic tumor cells accelerates tumor progression with PMN-MDSC infiltration

**Fig. 3:**
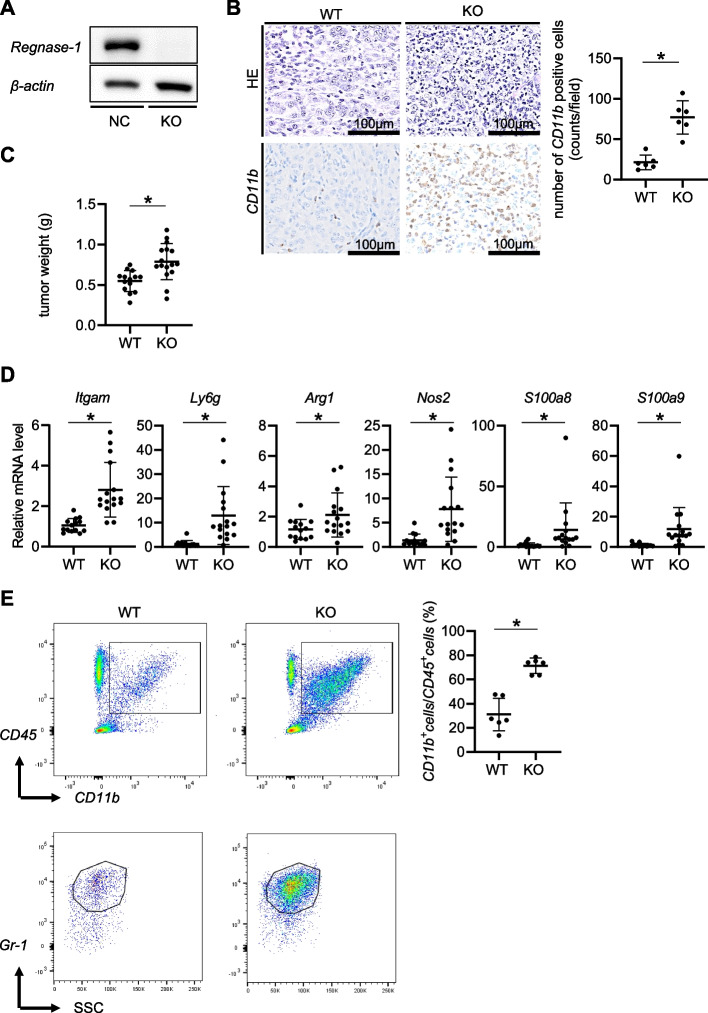
Regnase-1 deletion in pancreatic tumor cells accelerates tumor progression with PMN-MDSC infiltration. **A-E** Evaluation of phenotypes of orthotopic syngeneic tumors of WT or Rengase-1 KO pancreatic cancer cells established from pancreas-specific Kras and Tp53 mutant (KPC) mice. Regnase-1 and Actb protein levels in murine pancreatic cancer cell lines (**A**) (Representative figure of triplicated experiments). Representative images of HE staining (**B**, top) and CD11b immunostaining (**B**, bottom). The number of CD11b-positive cells (**B**, right**)** (*N* = 6 per group) and tumor weights (**C**) (*N* = 14–16 per group). The relative mRNA levels of *Itgam*, *Ly6g*, *Arg1*, *Nos2*, *S100a8*, and *S100a9* in the tumors (**D**) (*N* = 14–16 per group). Dot plots of CD45^+^CD11b^+^ cells (**E**, top) and CD45^+^CD11b^+^Gr-1 + cells (**E**, bottom) evaluated by flow cytometry. The proportion of CD11b + cells among CD45^+^ cells (**E**, right**)** (*N* = 6 per group). Student's t test was used to evaluate the differences between the two groups. *P < 0.05, scale bars: 100 μm (insets)

To further study the role of Regnase-1 in PDAC progression, we used a syngeneic orthotopic pancreatic cancer model. We first disrupted Regnase-1 by clustered regularly interspaced short palindromic repeats (CRISPR)/Cas9 in murine pancreatic cancer cells (KPCs) derived from KPC mice [16] (Fig. 3A). Regnase-1 deletion in KPCs significantly accelerated orthotopic tumor growth in immunocompetent wild-type mice and shortened their survival with marked infiltration of CD11b^+^ cells (Fig. 3B, C, Supplementary Fig. 6). Gene expression analysis showed significant elevation of the PMN-MDSC markers *Itgam* and *Ly6g*, together with the MDSC-derived cytokines/chemokines *Arg1*, *Nos*, *S100a8* and *S100a9,* in Regnase-1-deficient KPC-derived tumors compared to Regnase-1-proficient KPC-derived tumors (Fig. 3D). Flow cytometry confirmed the increase in CD11b^+^Gr1^+^ PMN-MDSCs in the Regnase-1-deficient KPC-derived tumors (Fig. 3E). We also showed the high levels of *Arg1*, *Nos*, *S100a8* and *S100a9,* in the sorted CD11b^+^ cells (Supplementary Fig. 7). These data suggested that Regnase-1 deletion in tumor cells accelerated PDAC progression with PMN-MDSC infiltration.

### Regnase-1 downregulation in pancreatic tumor cells increases the number of intratumor CD11b^+^ MDSCs, accelerating tumor progression

**Fig. 4:**
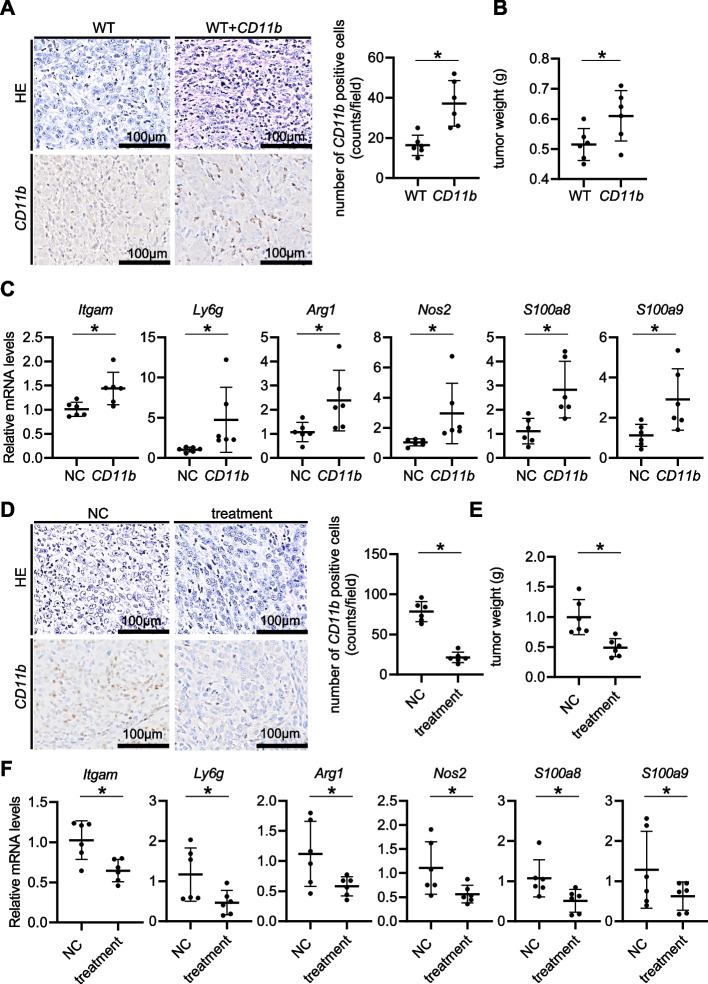
Regnase-1 downregulation in pancreatic tumor cells increases the number of intratumoral CD11b^+^ MDSCs, accelerating tumor progression. **A-D** Evaluation of phenotypes of orthotopic syngeneic tumors of WT murine pancreatic cancer cells with or without coinjection of CD11b^+^ cells obtained from pancreatic tumors in pancreas-specific Kras mutant Regnase-1 knockout (PKR) mice. Representative images of HE staining and CD11b immunostaining (**A**, left). The number of CD11b-positive cells (**A**, right) (*N* = 6 per group) and tumor weights (**B**) (*N* = 6 per group). The relative mRNA levels of *Itgam*, *Ly6g*, *Arg1*, *Nos2*, *S100a8*, and *S100a9* (**C**) (*N* = 6 per group). **D-F** Evaluation of phenotypes of orthotopic syngeneic tumors of Regnase-1-deleted murine pancreatic cancer cells with or without depletion of CD11b^+^ cells upon anti-Gr-1 antibody treatment. Representative images of HE staining and CD11b immunostaining (**D**, left). The number of CD11b-positive cells (**D**, right) (*N* = 6 per group) and tumor weights (**E**) (*N* = 6 per group). The relative mRNA levels of *Itgam*, *Ly6g*, *Arg1*, *Nos2*, *S100a8*, and *S100a9* (**F**) (*N* = 6 per group). Student's t test was used to evaluate differences between 2 groups. One-way ANOVA with Tukey's post hoc test was used to compare differences among 4 groups. *P < 0.05, scale bars: 100 μm (insets)

To evaluate the involvement of CD11b^+^ PMN-MDSCs induced by Regnase-1 loss in PDAC progression, we first isolated CD11b^+^ cells from the orthotopic tumors of Regnase-1-deficient KPCs and performed syngeneic orthotopic transplantation of Regnase-1-proficient KPCs with or without coinjection of isolated CD11b^+^ cells. Coinjection of isolated CD11b^+^ cells significantly accelerated orthotopic tumor growth of Regnase-1-proficient KPCs with an increase in the intratumor infiltration of CD11b^+^ MDSCs (Fig. 4A, B). We also confirmed the significant upregulation of a variety of MDSC markers, including *Itgam, Ly6g, Arg1, Nos2, S100a8*, and *S100a9,* in the tumors from Regnase-1-proficient KPCs coinjected with CD11b^+^ cells (Fig. 4C). Conversely, depletion of CD11b^+^ cells with Gr-1 antibody treatment significantly suppressed orthotopic tumor growth of Regnase-1-deficient KPCs (Fig. 4D, E). CD11b^+^-depleted tumors showed significant downregulation of MDSC markers, including *Itgam, Ly6g, Arg1, Nos2, S100a8*, and *S100a9* (Fig. 4F). Taken together, these findings suggested that the increase in intratumoral CD11b^+^ MDSCs may be responsible for the acceleration of pancreatic tumor progression in the absence of Regnase-1.

### IL-1β-induced Regnase-1 downregulation may produce a variety of cytokines/chemokines important for the intratumor increase in MDSCs

**Fig. 5:**
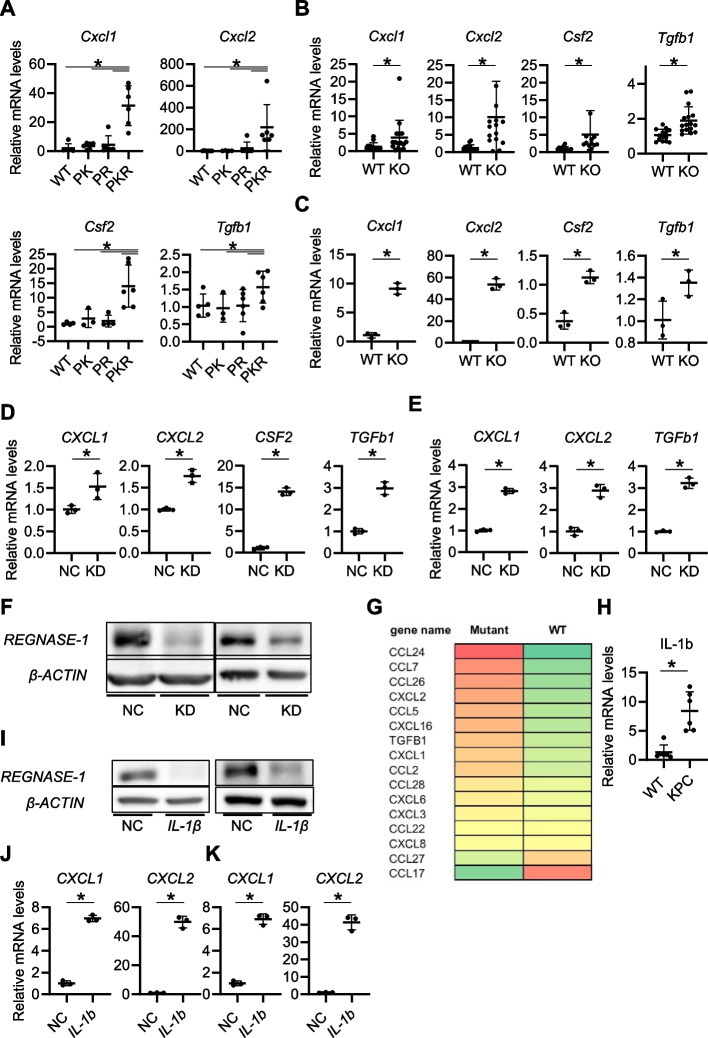
IL-1β-induced Regnase-1 downregulation may produce a variety of cytokines/chemokines important for the intratumor increase in MDSCs. **A** Relative mRNA levels of *Cxcl1*, *Cxcl2*, *Csf2*, and *Tgfb1* in pancreatic tissue of WT mice, pancreas-specific Regnase-1 knockout (PR) mice, pancreas-specific Kras mutant (PK) mice, and pancreas-specific Kras-mutant Regnase-1 knockout (PKR) mice at 24 weeks of age (*N* = 6 per group). **B** Relative mRNA levels of *Cxcl1*, *Cxcl2*, *Csf2*, and *Tgfb1* in orthotopic syngeneic tumors of WT or Regnase-1 KO pancreatic cancer cells (*N* = 14–16 per group). **C** Relative mRNA levels of *Cxcl1*, *Cxcl2*, *Csf2*, and *Tgfb1* in WT and Regnase-1 KO murine pancreatic cancer cell lines (*N* = 3 per group, experiments were performed triplicate). **D-E **Relative mRNA levels of *CXCL1*, *CXCL2*, *CSF2*, and *TGFB1* in human pancreatic cancer cell lines, Panc-1 (**D**) and MiaPaCa2 (**E**), upon knockdown (KD) of Regnase-1 by siRNA (*N* = 3 per group, experiments were performed triplicate). **F** Regnase-1 and ACTB protein levels in human pancreatic cancer cell lines, Panc-1 (left) and MiaPaCa2 (right), upon knockdown (KD) of Regnase-1 by siRNA. **G** Heatmap of relative expression levels of representative genes bound to Regnase-1 evaluated by RIP-seq between cells expressing wild-type Regnase-1 or mutant (D141N) Regnase-1 lacking RNase activity. **H** Relative *Il1b* mRNA levels **i**n the pancreas of WT mice and pancreatic tumors of pancreas-specific Kras- and Tp53-mutant (KPC) mice (*N* = 6 per group). **I** Regnase-1 and ACTB protein levels in human pancreatic cancer cell lines, Panc-1 (left) and MiaPaCa2 (right), with or without IL-1β stimulation. **J**,** K** Relative mRNA levels of *CXCL1* and *CXCL2* in the human pancreatic cancer cell lines Panc-1 (**J**) and MiaPaCa2 (**K**) with or without IL-1β stimulation (*N* = 3 per group, experiments were performed triplicate). Student's t test was used to evaluate differences between 2 groups. One-way ANOVA with Tukey's post hoc test was used to compare differences among 4 groups. *P < 0.05

We next investigated the mechanisms of MDSC increase in the Regnase-1-deficient tumors. Among a variety of chemokines/cytokines known to recruit and/or generate MDSCs in tumor sites, we found significant elevations in *Cxcl1*, *Cxcl2*, *Csf2*, and *Tgfb1* in the pancreatic tumor tissues of PKR mice (Fig. 5A). Upregulation of these genes was also observed in the Regnase-1-deficient KPC-derived orthotopic tumors compared to the Regnase-1-proficient KPC-derived orthotopic tumors (Fig. 5B). We thus examined the direct relationship between Regnase-1 and these genes in the cancer cells. We found that CRISPR-mediated Regnase-1 deletion significantly upregulated the mRNA expression of *Cxcl1*, *Cxcl2*, *Csf2*, and *Tgfb1* in KPC cells (Fig. 5C). We also confirmed that siRNA-mediated Regnase-1 inhibition significantly upregulated the mRNA expression of these genes in 2 human PDAC cell lines, Panc-1 and MiaPaCa-2 (Fig. 5D-F). These data suggested that Regnase-1 may negatively regulate these genes in pancreatic cancer cells. Because Regnase-1 functions to bind and directly degrade mRNA of targets, we aimed to comprehensively screen direct targets of Regnase-1 in Panc-1 cells. To this end, we performed RIP-seq using Regnase-1-deficient Panc-1 cells with transient expression of wild-type or mutant Regnase-1 protein (lack of the mRNA degrading capacity). We found higher levels of Cxcl1, Cxcl2, and TGFβ mRNAs bound to mutant Regnase-1 protein than those bound to wild-type Regnase-1 protein (Fig. 5G), suggesting that Regnase-1 may directly bind to and degrade these cytokines/chemokines in pancreatic tumor cells. Taken together these data suggested that Regnase-1 negatively regulated a variety of cytokines/chemokines important for the increase in MDSCs in tumor sites.

We then searched for the factors responsible for the Regnase-1 downregulation observed in the pancreatic tumors of KPC mice (Fig. 2C). IL-1β and IL-17A are known to negatively regulate Regnase-1 [18, 19] and we found significant upregulation of IL-1β but did not detect IL-17A expression in the tumor sites (Fig. 5H, Supplementary Fig. 8). Indeed, recombinant IL-1β treatment downregulated Regnase-1 expression with significant elevation of *CXCL1* and *CXCL2* levels in 2 human PDAC cell lines (Fig. 5I-K). Taken together, our findings suggested that IL-1β-induced Regnase-1 downregulation may produce a variety of cytokines/chemokines important for the intratumor MDSC increase, promoting pancreatic tumors. Because IL-1β is a major regulator of the expression of a myriad of proinflammatory cytokines such as IL-6 that is the main regulatory cytokine of MDSC activity and involved in the PDAC pathogenesis [20, 21]. We thus further evaluated the involvement of IL-6 in Regnase-1 regulation by IL-1β. We found a significant upregulation of IL-6 in the KPC tumors and IL-1β treatment significantly upregulated IL-6 in the Panc-1 cells (Supplementary Fig. 9A, B). However, neither downregulation of Rengase-1 nor upregulation of CXC chemokines upon IL-1β treatment was not suppressed by the neutralization of IL-6 (Supplementary Fig. 9C, D). Therefore, IL-6 may not be involved in the regulation of Regnase-1/CXC chemokine by IL-1β.

### Suppression of cytotoxic T lymphocytes (CTLs) by CD11b^+^ MDSCs is responsible for the acceleration of tumor progression by Regnase-1 downregulation

**Fig. 6:**
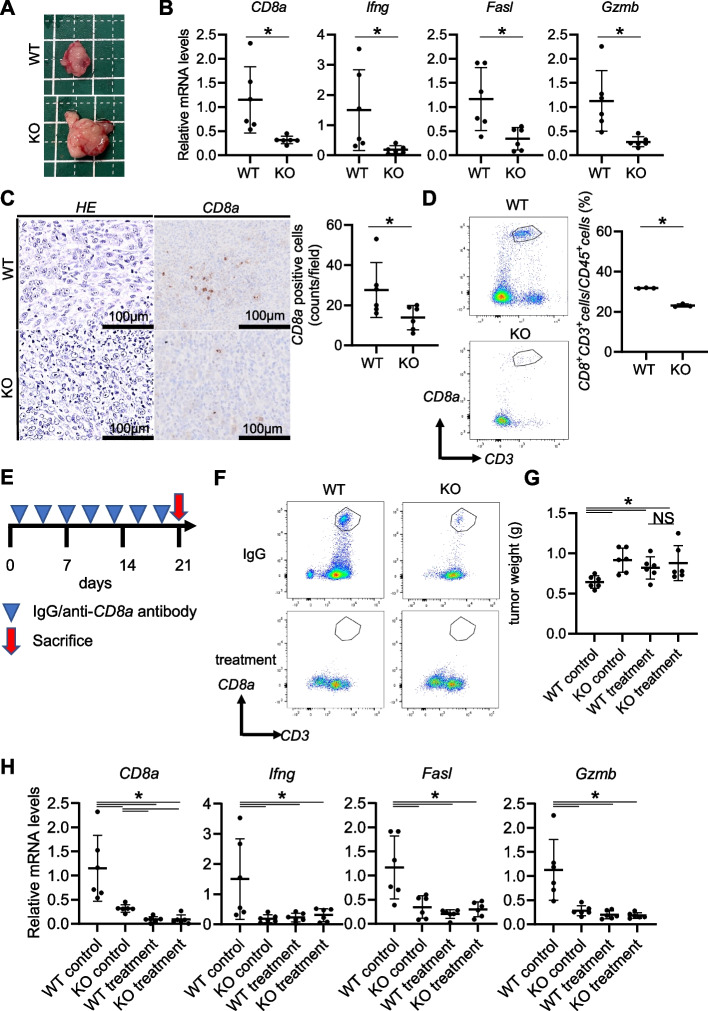
Suppression of CTLs by CD11b^+^ MDSCs is responsible for the acceleration of tumor progression by Regnase-1 downregulation. **A-D** Evaluation of phenotypes of orthotopic syngeneic tumors of WT or Regnase-1 KO murine pancreatic cancer cells. Representative macro images of pancreatic tumors (**A**). Relative mRNA levels of *Cd8a*, *Ifng*, *Fasl*, and *Gzmb* (**B**) (*N* = 6 per group). Representative images of HE (**C**, left panel) and CD8a immunostaining (**C**, right panel) and the number of CD8-positive cells (**C**, right) (*N* = 6 per group). Dot plots of CD3^+^CD8^+^ cells evaluated by flow cytometry (**D**, left) and the proportion of CD8 + cells among CD45^+^ cells (**D**, right) (*N* = 3 per group). **E–H** Evaluation of phenotypes of orthotopic syngeneic tumors of WT or Regnase-1-KO murine pancreatic cancer cells with or without depletion of CD8^+^ cells upon anti-CD8a antibody or IgG treatment. Experimental schematic (**E**). Dot plots of CD3^+^ and CD8.^+^ cells in WT or Regnase-1-KO syngeneic tumors upon anti-CD8a antibody or IgG treatment evaluated by flow cytometry (**F**). Tumor weights (**G**) (*N* = 6 per group). The relative mRNA levels of *Cd8a*, *Ifng*, *Fasl*, and *Gzmb* (**H**) (*N* = 6 per group). Student's t test was used to evaluate differences between 2 groups. One-way ANOVA with Tukey's post hoc test was used to compare differences among 4 groups. *P < 0.05, scale bars: 100 μm (insets)

Finally, we aimed to further clarify the mechanisms of MDSC-mediated PDAC progression in the absence of Regnase-1. Among a variety of immune cells in the tumor microenvironment, we found that the number of CD8^+^ CTLs was significantly lower in Regnase-1-deficient orthotopic KPC tumors than in Regnase-1-proficient tumors (Fig. 6A-D). Accordingly, the expression levels of *Ifng*, *Fasl*, and *Gzmb*, all of which are important CTL-derived cytotoxic factors, were also significantly decreased in the Regnase-1-deficient orthotopic KPC tumors (Fig. 6B). These data suggested MDSC-mediated suppression of antitumor immunity in the Regnase-1-deficient pancreatic tumors. We next depleted CD8^+^ T cells with an anti-CD8 antibody in orthotopic KPC tumors (Fig. 6E, F). The growth of Regnase-1-proficient orthotopic KPC tumors was significantly accelerated by the depletion of CD8^+^ T cells, suggesting the importance of CTL-mediated antitumor immunity in controlling pancreatic tumor growth (Fig. 6G). Importantly, in the absence of CD8^+^ T cells, the tumor-promoting effect of Regnase-1 disruption on orthotopic KPC tumors was diminished (Fig. 6G, H). These findings suggested that suppression of CTLs by CD11b^+^ MDSCs may be responsible for the acceleration of pancreatic tumor growth by Regnase-1 downregulation.

## Discussion

In this study, focusing on Regnase-1, an RNA-binding protein with endoribonuclease activity, we examined the clinical significance of Regnase-1 expression in PDAC patients and explores its functional roles using mouse models and cell lines. The fact that intratumor Regnase-1 levels were inversely associated with CD11b^+^ cell infiltration and clinical outcome in PDAC patients suggested tumor suppressive roles of Regnase-1 in human PDAC progression. This was also confirmed in the external dataset (Supplementary Fig. 10). However, since clinical data can only show correlation, we generated the Regnase-1 knockout mice/cell lines and demonstrated the causal relationship such that Regnase-1 downregulation recruits MDSCs and contributes to pancreatic cancer progression via mitigation of CTL-mediated antitumor immunity.

Inflammation plays an important role in pancreatic cancer [3]. Previous studies have shown that both Kras mutations and inflammation are essential for the development of pancreatic cancer [4]: inflammatory stimulation suppresses oncogene-induced cellular senescence in Kras mutant pancreatic cells, leading to Pancreatic intraepithelial neoplasia (PanIN) progression and pancreatic cancer [22]. In addition, several molecules were identified as inflammatory factors linking inflammation and pancreatic tumorigenesis. Chronic inflammation induces IL-6 secretion from myeloid cells and pancreatic stellate cells, which promotes pancreatic cancer cell proliferation and invasiveness [23]. The inflammatory cytokines TNF and IL-1α are also known to promote pancreatic cancer [24, 25]. Our study identified Regnase-1 as a new important player linking inflammation and pancreatic cancer progression. Pancreas-specific Regnase-1 deletion induced spontaneous pancreatitis and promoted Kras-driven pancreatic cancer in mice. Mechanistically, Regnase-1 deletion induced a variety of chemokines, recruiting MDSCs in the tumor microenvironment. We also found that the inflammatory cytokine IL-1β negatively regulated Regnase-1 in pancreatic tumor cells. Das S et al. recently reported that IL-1β promoted the activation and secretory phenotypes of quiescent PSCs, leading to the establishment of a protumorigenic microenvironment in PDAC [26]. Taken together, these findings illustrated the importance of IL-1β-mediated reshaping of the tumor microenvironment in PDAC promotion.

Regnase-1 is mainly localized in the endoplasmic reticulum or cytoplasm and has a CCCH-type zinc finger region, an RNase region, and a PilT N-terminus-like (PIN) domain [9]. Regnase-1 interacts with ribosomal proteins to degrade mRNAs undergoing translation. In particular, it binds to stem‒loop structures in the 3'-untranslated region (3'UTR) of mRNAs for inflammation-related molecules such as IL-6, IL-12B, PTGS2, Cxcl1, and Cxcl2 and regulates immune responses by destabilizing the mRNA [9]. In our study, RIP-seq analysis showed the direct negative regulation of a variety of important cytokines/chemokines by Regnase-1 in PDAC cells, including previously reported Cxcl1 and Cxcl2. During inflammation, inflammatory stimuli such as IL-1β and TLRs transduce the signaling pathway through MyD88 and activate the IKK complex. The activated IKK complex phosphorylates Regnase-1 and degrades it by ubiquitination. As a result, the production of inflammatory cytokines such as IL-6 increases and further accelerates the inflammatory response [10]. Indeed, as mentioned above, IL-1β-mediated Regnase-1 degradation was also observed in our study, which contributed to MDSC recruitment and PDAC progression. However, Regnase-1 is known to target and degrade its own mRNA. Recently, Tse KM et al. showed that the disruption of the stem‒loop structure of Regnase-1 released its autoinhibition [27]. These researchers succeeded in increasing Regnase-1 levels in various cells using a Regnase-1 stabilizer [27] and showed that Regnase-1 stabilizing agents alleviated autoimmune diseases such as acute respiratory distress syndrome and multiple sclerosis via suppression of excessive inflammation. Given that IL-1β-mediated degradation of Regnase-1 promoted PDAC in our study, Regnase-1 stabilizing agents could be used to suppress pancreatic cancer progression.

MDSCs are a heterogeneous population of immature bone marrow cells and are implicated in many pathological conditions, such as obesity, autoimmunity, chronic inflammation, trauma, and cancer progression [28, 29]. During cancer progression, MDSCs accumulate and proliferate in tumor sites, where they modulate host antitumor immune responses [30–33]. In general, MDSC-induced immunosuppression is mediated through the orchestration of multiple pathways, including direct cell-to-cell interactions and the production of immunosuppressive factors such as ARG1, NOS, S100A8, and S100A9, leading to cancer progression and interference with the efficacy of therapeutic agents such as immune checkpoint inhibitors [30–34]. Several studies have shown a positive correlation between MDSCs and PDAC progression [33, 35, 36], and MDSCs thus may pose a serious challenge to the treatment of PDAC. In this study, we showed that MDSCs played tumor-promoting roles in PDAC via the suppression of CTL-mediated antitumor immunity, and the elimination of MDSCs suppressed tumor growth in PDAC. These findings were consistent with previous reports [36–39]. Moreover, we first identified the novel regulatory role of Regnase-1 in MDSC recruitment in the pancreatic tumor site. Regnase-1 negatively regulates a variety of cytokines/chemokines important for the recruitment and education of MDSCs, including Cxcl1, Cxcl2, Csf2, and TGFβ. Although the potential of MDSCs as therapeutic targets for PDAC has been recognized, cancer therapies directly targeting MDSCs have yet to succeed [40]. Our current findings may identify an alternative strategy to suppress MDSCs by modulating Regnase-1, which could be a novel PDAC therapeutic.

There are the following limitations in this study: Lack of evaluation for phenotypes of Regnase-1 upregulation, lack of validation of some murine findings in human specimens, and the lack of evaluation of protein levels for some transcriptional changes.

## Conclusion

We have identified novel and important findings that IL-1b-mediated Regnase-1 downregulation induced MDSCs and promoted pancreatic cancer through the evasion of anticancer immunity. This mechanistic link between inflammation and PDAC progression may facilitate the development of therapeutics targeting inflammation-driven cancer progression in PDAC patients.

### Supplementary Information

**Additional file 1.**

**Additional file 2.**

## Data Availability

The datasets used and/or analysed during the current study are available from the corresponding author on reasonable request.

## Abbreviations

CEA: Carcinoembryonic antigen
CP: Chronic pancreatitis
CSF: Colony stimulating factor
CTLs: Cytotoxic T cells
Cxcl: C-X-C motif chemokine ligand
IHC: Immunohistochemistry
IL-1β: Interleukin-1β
KPC mice: Pancreas-specific KrasG12D mutant and Tp53 knockout mice
PMN-MDSC: Polymorphonuclear myeloid-derived suppressor cell
PKR mice: Pancreas-specific Regnase-1 knockout and KrasG12D mutant mice
PDAC: Pancreatic ductal adenocarcinoma
