# Supplementary material for: Regnase-1 downregulation promotes pancreatic cancer through myeloid-derived suppressor cell-mediated evasion of anticancer immunity
Source: J Exp Clin Cancer Res. 2023 Oct 9;42:262. doi: 10.1186/s13046-023-02831-w (PMC10561497; doi:10.1186/s13046-023-02831-w)
Supplement: Supplementary file 1 — Additional file 1. [file 13046_2023_2831_MOESM1_ESM.docx]

**Figure Legends**

**Supple Fig 1.** Raw western blot images.

**Supple Fig 2.** **(A-B)** Thirty-nine pancreatic cancer patients were classified into two groups according to the Regnase-1 immunostaining scores in pancreatic tumors. Representative images of CD3 (left) and CD20 (right) staining of pancreatic tumors in pancreatic cancer patients **(A)**. Correlation between Regnase-1 immunostaining scores and CD3^+^ (left) and CD20^+^ (right) cell counts of pancreatic tumors in pancreatic cancer patients **(B)**. The Pearson product-moment correlation coefficient was used to determine the correlation coefficient. Scale bars: 200 μm (insets).

**Supple Fig 3.** Regnase-1 and Actb protein levels in wild-type (WT) mice and pancreas-specific Regnase-1 knockout (PR) mice.

**Supple Fig 4.** Relative mRNA levels of *Ccl2, Cox2, Cxcl12, Cxcr2, Ccr4, Il6st,* and *Il10* in pancreatic tissue of WT mice, pancreas-specific Regnase-1 knockout (PR) mice, pancreas-specific Kras-mutant (PK) mice, and pancreas-specific Kras-mutant Regnase-1 knockout (PKR) mice at 4 weeks of age (N=6 per group). One-way analysis of variance followed by Tukey's post hoc test was used to compare differences between the four groups. *: P < 0.05,

**Supple Fig 5.** Dot plots and percentage of Ly6g^+^Ly6c^low^ cells and Ly6g^-^Ly6c^+^ cells among CD45^+^CD11b^+^ cells in the spleen of WT mice **(left)** and in the tumor of pancreas-specific Kras-mutant Regnase-1 knockout (PKR) mice **(right)** evaluated by flow cytometry.

**Supple Fig 6.** Survival of the mice with orthotopic syngeneic tumors of WT or Rengase-1 KO pancreatic cancer cells established from pancreas-specific Kras and Tp53 mutant (KPC) mice (N=10 each). The percentage of the mice that survived at 27 days after tumor inoculation was shown (70% [7 out of 10] in the WT group and 20% [2 out of 10] in the KO group). * P<0.05 by chi-square test.

**Supple Fig 7.** Relative mRNA levels of *Itgam, Ly6g, Arg1, Nos2, S100a8,* and *S100a9* in CD11b+ or CD11b^-^ cells isolated from the Regnase-1-deficient orthotopic tumors (N=5 per group). Student's t test was used to evaluate the differences between the two groups. *P <0.05.

.

**Supple Fig 8.** Il1b and Actb protein levels in the pancreas of WT mice and and pancreatic tumors of pancreas-specific Kras- and Tp53-mutant (KPC) mice.

**Supple Fig 9.** **(A)** Relative *Il6* mRNA levels **i**n the pancreas of WT mice and pancreatic tumors of pancreas-specific Kras- and Tp53-mutant (KPC) mice (N=3 per group). **(B)** Relative mRNA levels of *IL6* in the human pancreatic cancer cell lines Panc-1 **(left)** and MiaPaCa2 **(right)** with or without IL-1β stimulation (N=3 per group). **(C)** Regnase-1 and ACTB protein levels in Panc-1 cells with or without IL-1β stimulation and/or anti-IL-6 antibody (Tocilizumab, Selleck, 5 μg/ml). **(D)** Relative mRNA levels of *CXCL1* and *CXCL2* in Panc-1 cells with or without IL-1β stimulation and/or anti-IL-6 antibody (Tocilizumab, Selleck, 5 μg/ml). (N=3 per group). Student's t test was used to evaluate differences between 2 groups. One-way ANOVA with Tukey's post hoc test was used to compare differences among 4 groups. *P <0.05.

**Supple Fig 10.** Correlation between mRNA levels of REGNASE-1 (ZC3H12A) and the clinical outcomes of pancreatic ductal adenocarcinoma (PDAC) patients was evaluated using publicly available expression datasets in the Kaplan–Meier Plotter database. Kaplan–Meier curves of disease free survival of 150 PDAC patients stratified based on the median value of mRNA levels of REGNASE-1.
