# Supplementary material for: Regnase-1 downregulation promotes pancreatic cancer through myeloid-derived suppressor cell-mediated evasion of anticancer immunity
Source: J Exp Clin Cancer Res. 2023 Oct 9;42:262. doi: 10.1186/s13046-023-02831-w (PMC10561497; doi:10.1186/s13046-023-02831-w)
Supplement: Supplementary file 2 — Additional file 2. [file 13046_2023_2831_MOESM2_ESM.zip › OkabeSuppleFigure_20230906.pdf]

Figure 3A

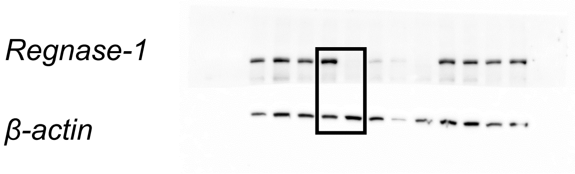

Figure 5F

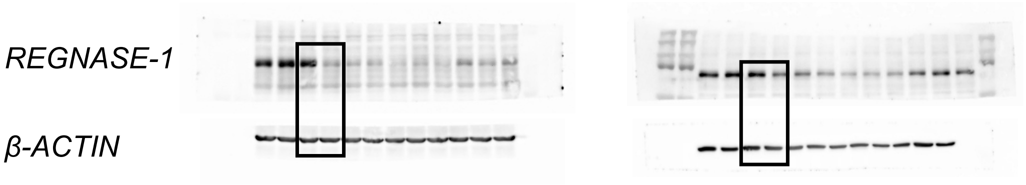

Figure 5I

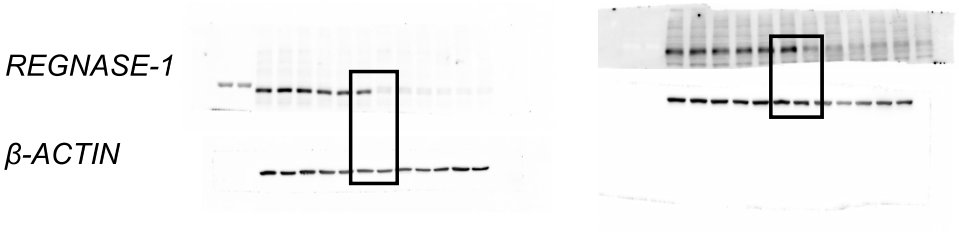

Supple Figure 3

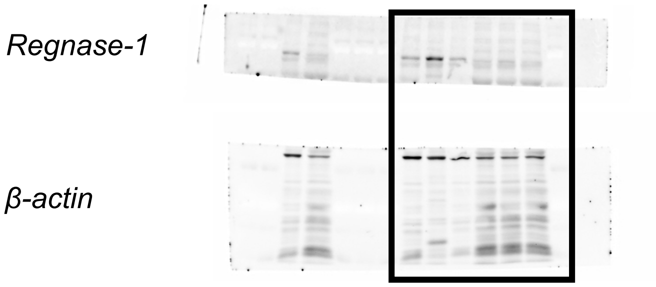

Supple Figure 8

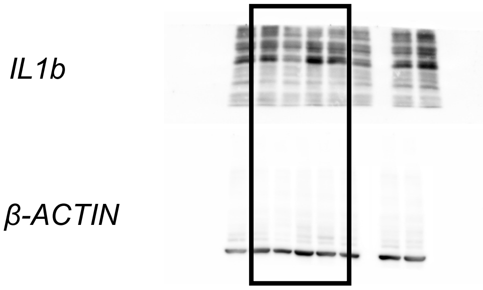

Supple Figure 9C

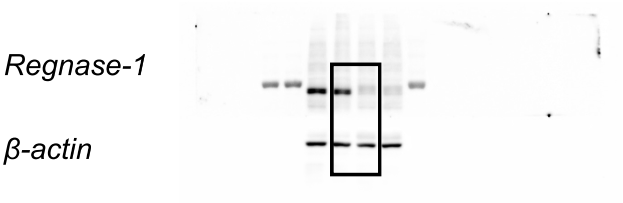

SuppleFigure 1

A

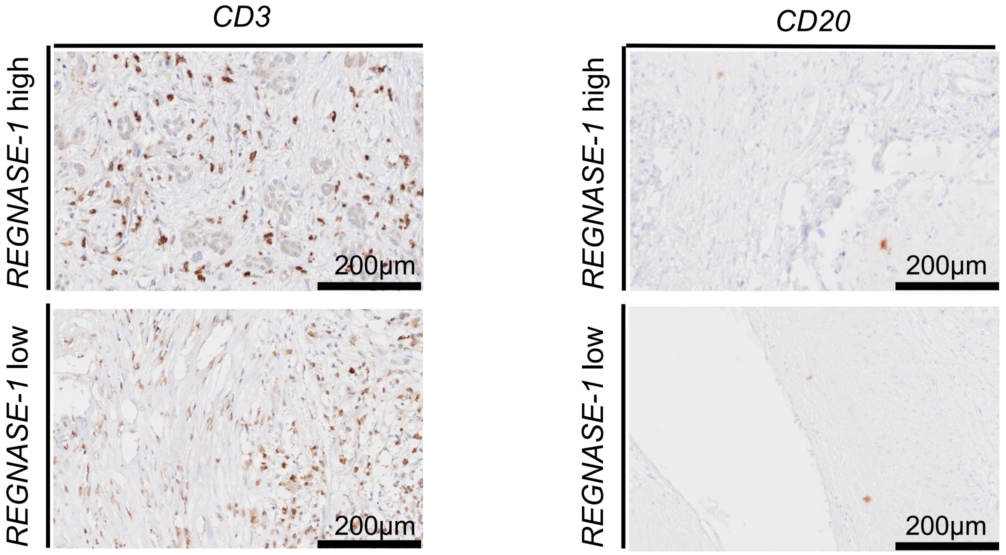

B

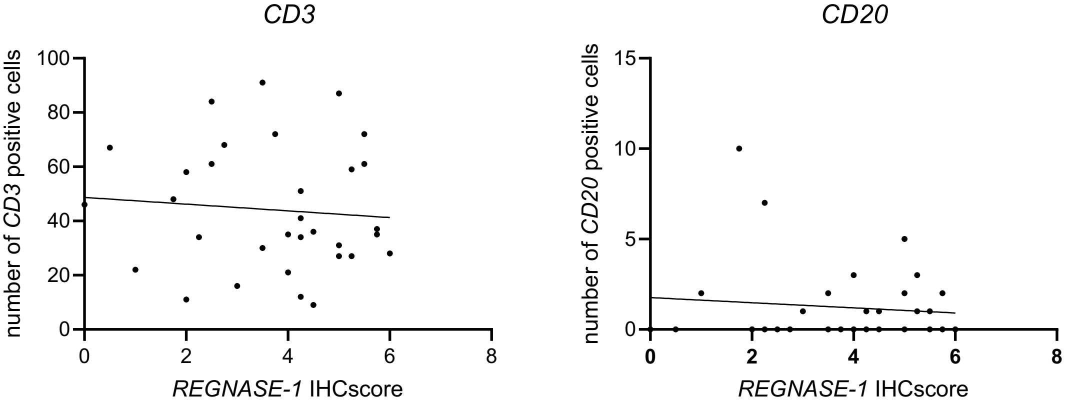

SuppleFigure 2

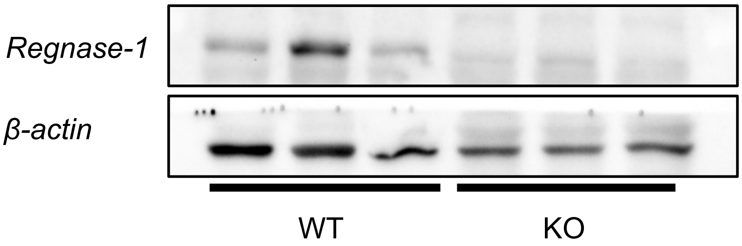

SuppleFigure 3

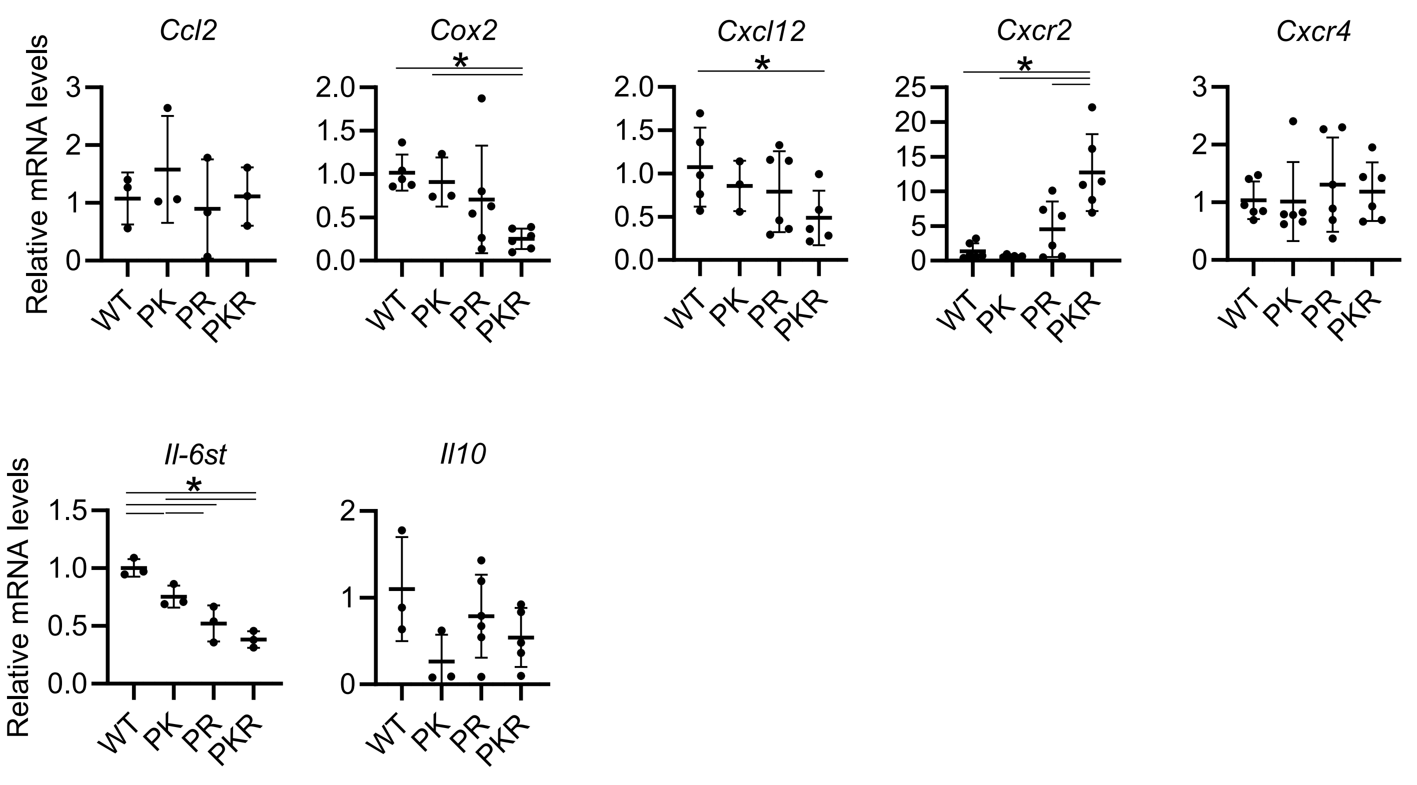

SuppleFigure 4

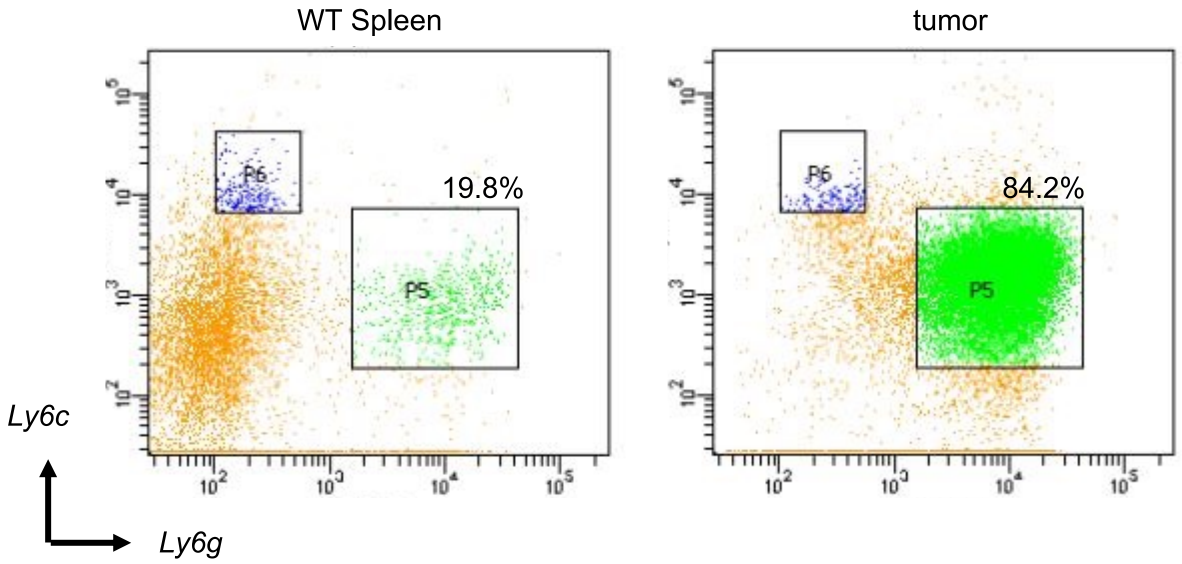

SuppleFigure 5

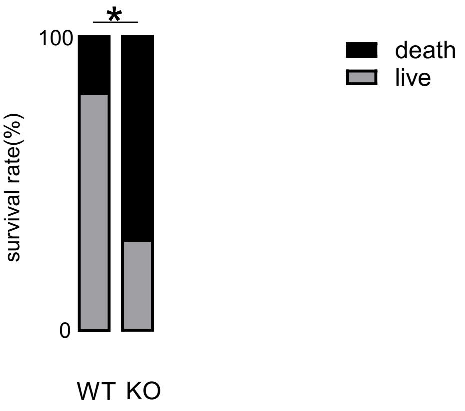

SuppleFigure 6

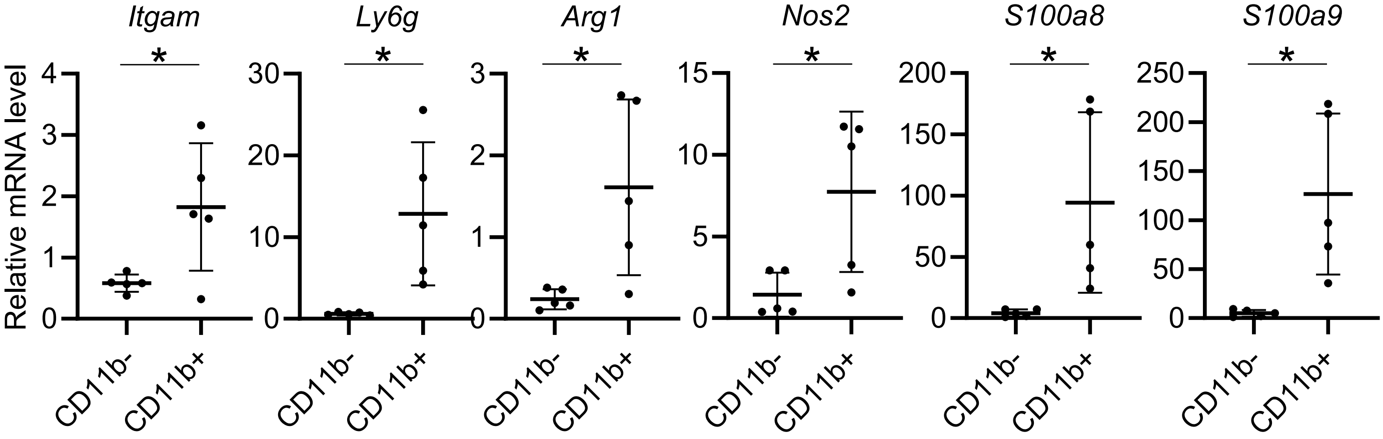

SuppleFigure 7

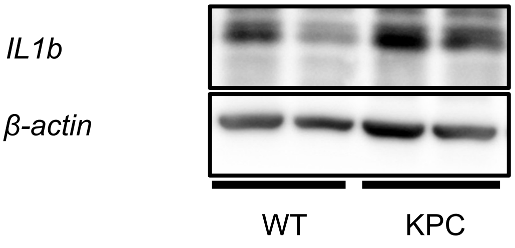

SuppleFigure 8

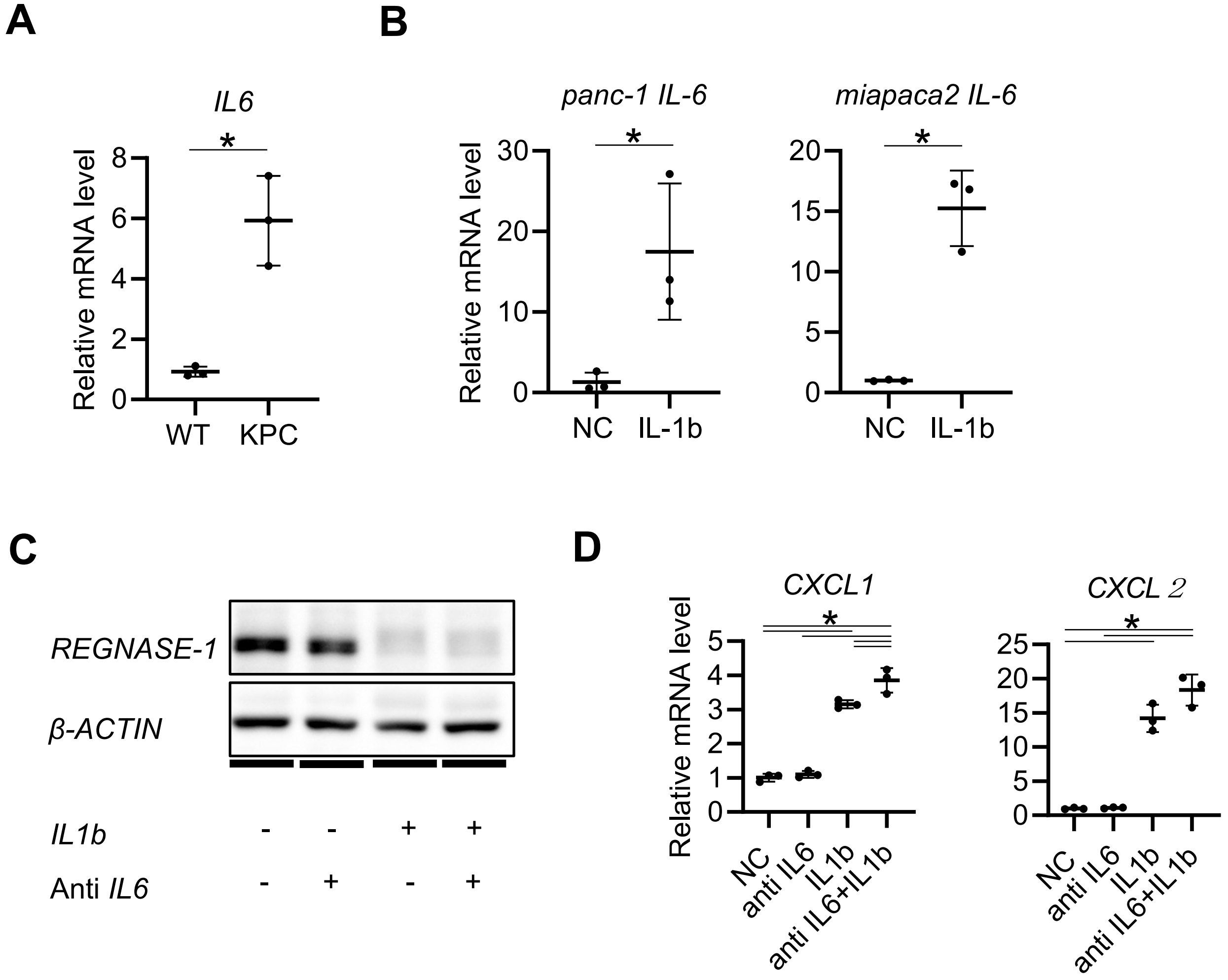

SuppleFigure 9

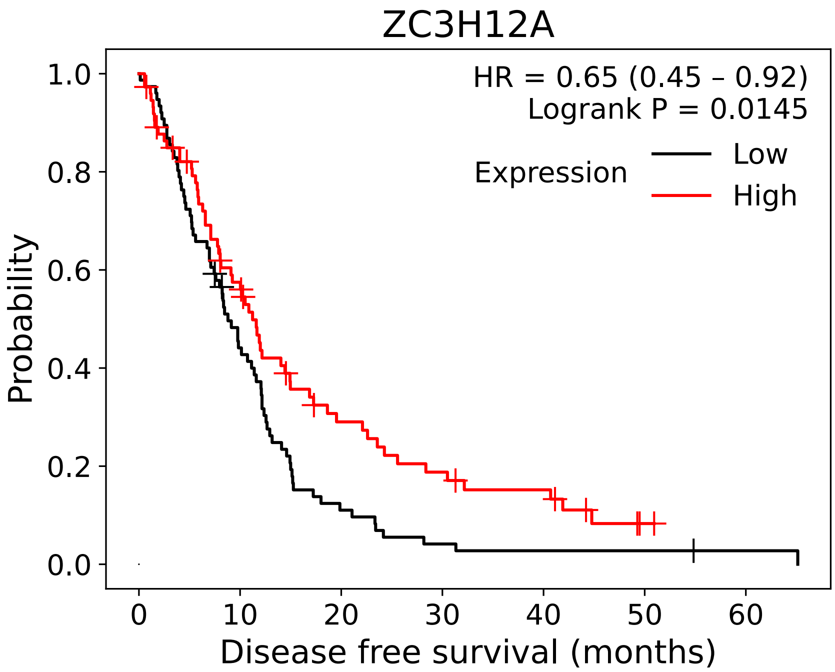

At risk							
Low	76	32	8	3	2	2	1
High	74	39	17	11	8	1	0

SuppleFigure10
